# Supplementary material for: Myc controls a distinct transcriptional program in fetal thymic epithelial cells that determines thymus growth
Source: Nat Commun. 2019 Dec 2;10:5498. doi: 10.1038/s41467-019-13465-y (PMC6889275; doi:10.1038/s41467-019-13465-y)
Supplement: Supplementary file 1 — Supplementary Information [file 41467_2019_13465_MOESM1_ESM.pdf]

Supplementary Information

Myc Controls A Distinct Transcriptional Program in Fetal Thymic Epithelial Cells That Determines Thymus Growth. Cowan et al.

Supplementary Figure 1

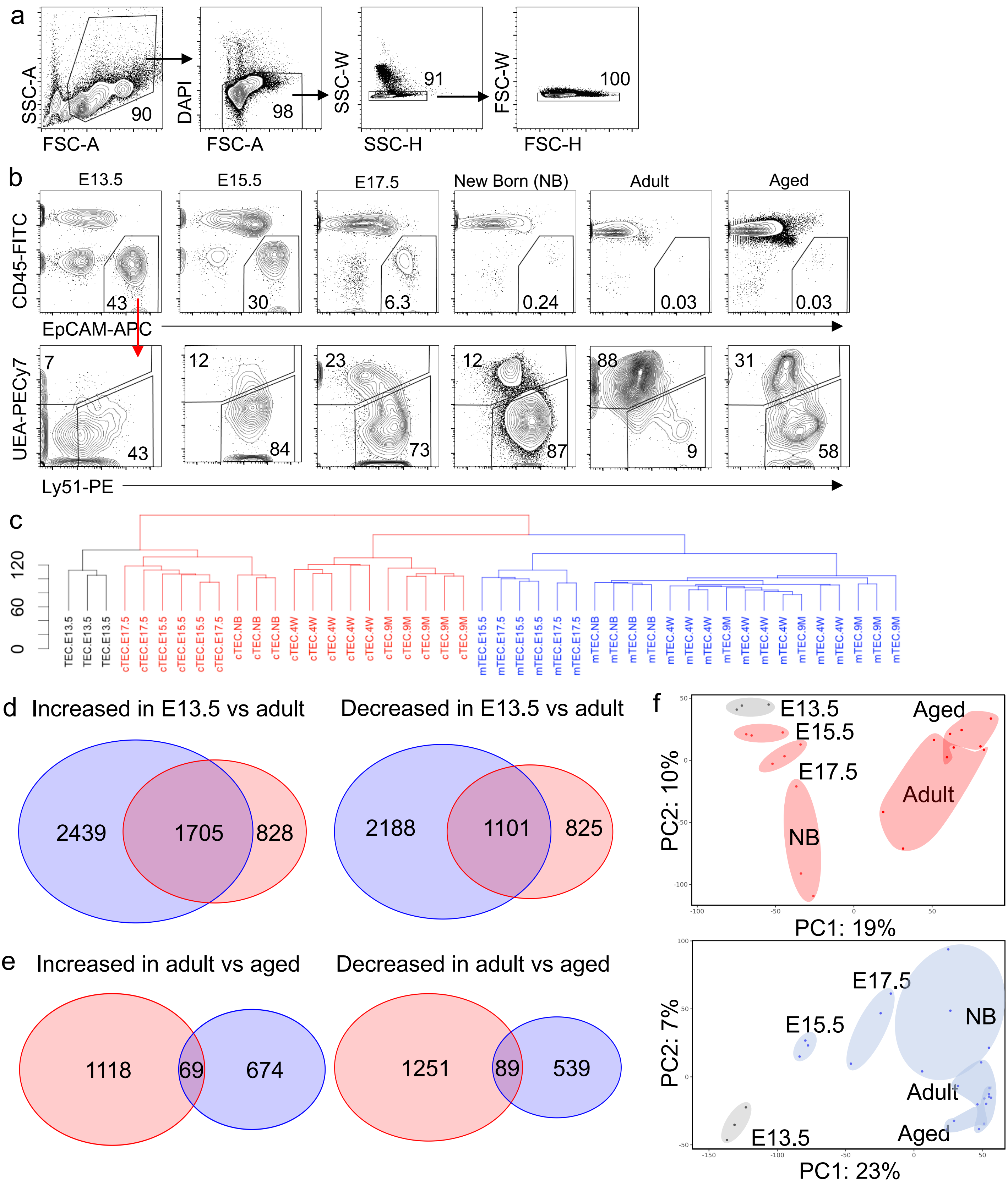

g

cTEC: Increased in adult vs aged

Pathway	P value
DANG Myc Targets UP	3.8E-06
Hallmark Myc Targets	4.3E-08
Proliferation	5.6E-15
DANG Bound by Myc	9.3E-06
GO Cell division	3.3E-18
GO G2-M Cell cycle	1.1E-08

cTEC: Decreased in adult vs aged

IFNγ Responses	2.2E-4
Cell migration	6E-05
Epithelium development	1.5E-06

mTEC: Increased in adult vs aged

Pathway	P value
Mitochondrial respiratory chain	9.3E-05
Mitochondrial respiratory chain complex III	7.6E-04
Oxidative phosphorylation	2.3E-03
Cell cycle genes in I.R response	3.5E-03

mTEC: Decreased in adult vs aged

Receptor activity	2.9E-04
Regulation of membrane potential	3.5E-04

Supplementary Figure 1: Transcriptional profiling of thymic epithelial cells from embryonic to adult stages in development. (a) Representative flow plots of live/dead and doublet discrimination. (b) Representative flow plots displaying frequencies of $CD45^{-}EpCAM^{+}$ total TEC (above) or $CD45^{-}EpCAM^{+}Ly51^{+}UEA^{-}$ cTEC and $CD45^{-}EpCAM^{+}Ly51^{-}UEA^{+}$ mTEC populations (below) at indicated ages. (c) A minimum spanning tree for each individual population that was analyzed by bulk RNA-seq. Total TEC (black lines) or cTEC (red) and mTEC (blue) populations isolated at time points indicated.(d) Venn diagrams displaying the number of differentially expressed genes increased (left) or decreased (right) in E13.5 TEC versus adult cTEC (red) or mTEC (blue) populations, derived using 2-fold change and $p<0.05$. (e) A principle component analysis of either E13.5 TEC (black) and all cTEC samples (red) (above), or E13.5 TEC (black) and all mTEC samples (blue) (below), where each data point is an individual sample and the shaded ovals identify replicate populations. (f) Venn diagrams displaying the number of differentially expressed genes increased (left) or decreased (right) in adult TEC versus aged TEC populations; cTEC (red) or mTEC (blue), derived using 2-fold change and $p<0.05$. (g) Tables of pathway enrichment analysis performed on the top 1000 genes (sorted by t-statistic) increased (above) or decreased (below) in adult cTEC (left) or mTEC (right) compared to aged TEC populations, with pathways on left and p values on right.

Supplementary Figure 2

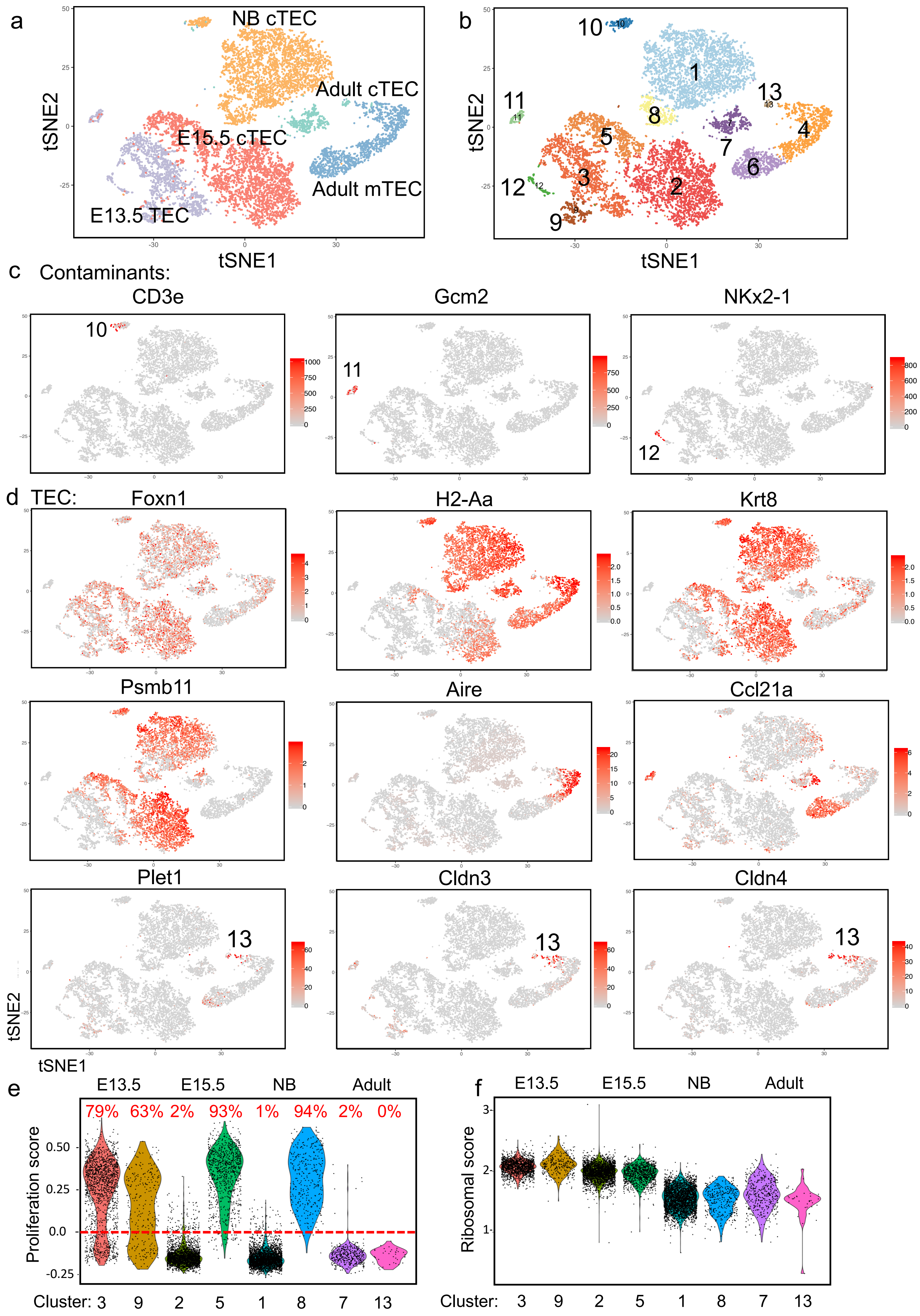

Supplementary Figure 2: Single cell transcriptome analysis of TEC from fetal to adult stages in development. (a-b) A tSNE plot of scRNA-seq data of individual populations isolated at indicated time point, colored and clustered by identity (a) or gene expression similarity, at a resolution of 0.4 (b). Each dot represents one cell. (c-d) tSNE plots of expression of individual genes indicated. Contaminates were removed from analysis (based on their expression of: CD3e for thymocytes (cluster 10) Gcm2 and Ccl21a expression for parathyroid (cluster 11) and NKX2-1 for thyroid (cluster 12). (e) A violin plot of the proliferation score applied to the indicated cluster number labelled in b, from the indicated developmental time point. Each dot represents one cell. The red line separates cells based on their proliferation score, high score above the line and low score below the line, percentages of cells above the line are indicated in red. (f) A violin plot of the ribosomal score applied to the indicated cluster number labelled in b, from the indicated developmental time point.

Supplementary Figure 3

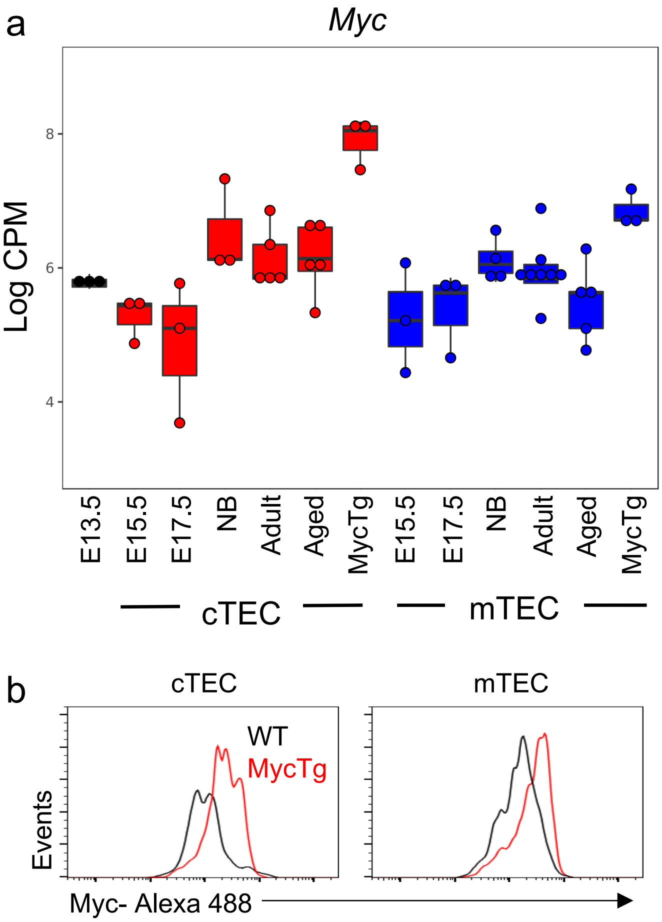

Supplementary Figure 3: The Myc transgene increased levels of Myc mRNA and Myc protein in adult TEC. (a) Box plot of Myc transcript levels from bulk RNA-seq data on WT TEC (black), cTEC (red), and mTEC (blue), isolated from indicated time points and adult FoxN1MycTg (striped bar). The normalized *Myc* counts for WT samples refer to mouse *Myc*, and for the FoxN1MycTg mouse, to human *MYC* counts. Box plots show the median and the interquartile range, with dots representing the individual datapoints. (b) Intracellular antibody staining for Myc protein in TEC of adult WT (black line) and adult FoxN1MycTg TEC (red line) displayed as a histogram.

Supplementary Figure 4

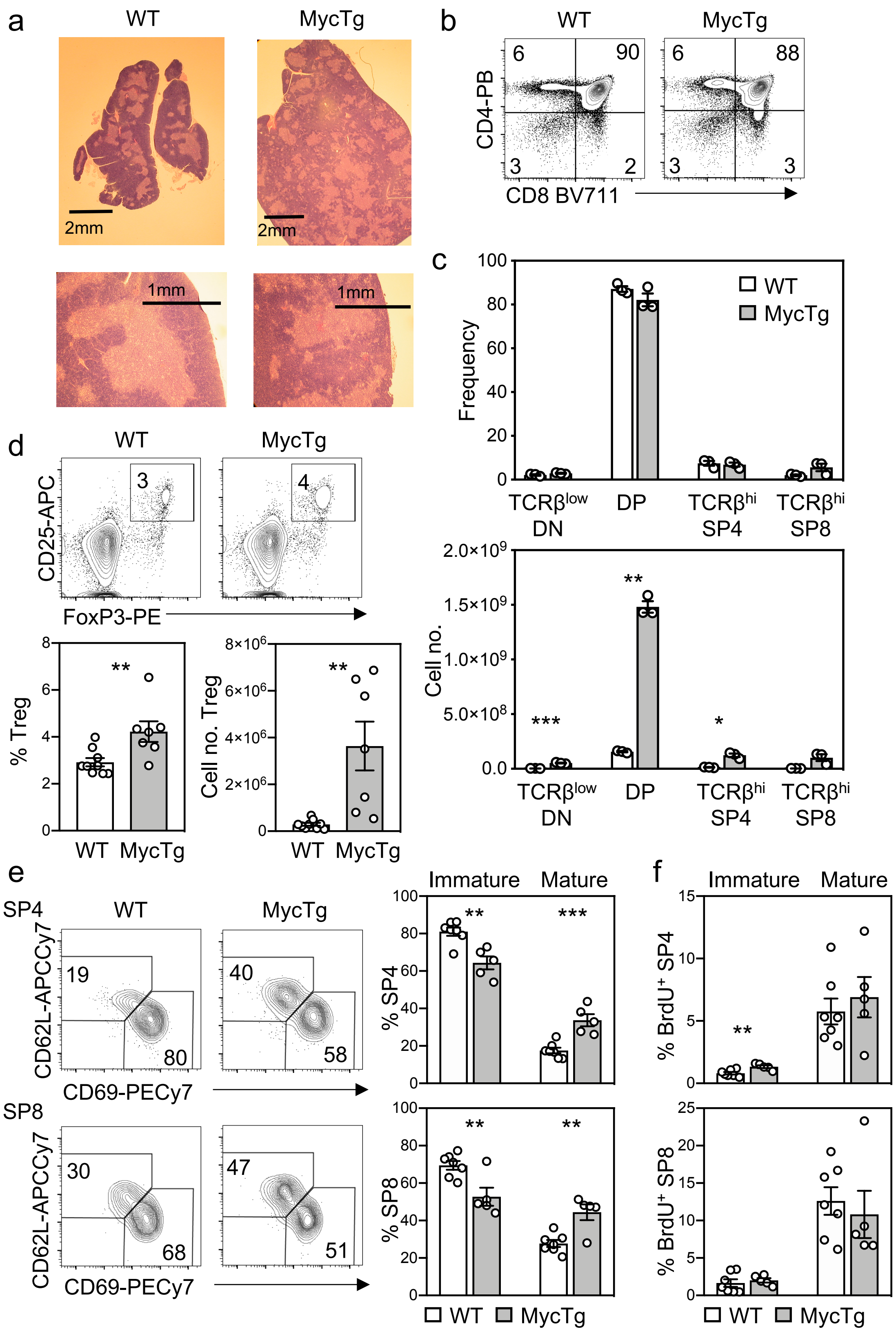

Supplementary Figure 4: Thymocytes development in the FoxN1MycTg mice. (a) Hematoxylin and eosin staining of PFA fixed thymus tissues from adult WT or FoxN1MycTg adult mice. Black lines display scale bars, 2mm in pictures above, 1mm in pictures below. (b) Representative flow plots of thymocyte analysis for CD4 versus CD8 expression on WT and FoxN1MycTg adult mice. (c) Representative bar graphs of the frequency (above) or numbers (below) of $TCRb^{low}$ $CD4^{-}CD8^{-}$. $CD4^{+}CD8^{+}$, $TCRb^{high}CD4^{+}$ $CD8^{-}$ and $TCRb^{high}CD4^{-}CD8^{+}$ populations from WT or FoxN1MycTg mice. (d) Representative flow plots of CD25 versus FoxP3 expression or bar graphs of the frequency (left) and numbers (right) of $CD25^{+}$ $FoxP3^{+}$ $TCRb^{high}CD4^{+}$ $CD8^{-}$ T Regulatory cells from WT or FoxN1MycTg mice. (e) Representative flow plots or bar graphs of the frequency of immature $CD69^{+}$ $CD62L^{-}$ or mature $CD69^{-}$ $CD62L^{+}$ $TCRb^{high}CD4^{+}$ $CD8^{-}$ (above) or $TCRb^{high}CD4^{-}$ $CD8^{+}$ (below) populations from WT (white) or FoxN1MycTg (gray) mice. (f) Bar graphs of the frequency of $BrdU^{+}$ immature $CD69^{+}$ $CD62L^{-}$ or mature $CD69^{-}$ $CD62L^{+}$ $TCRb^{high}CD4^{+}$ $CD8^{-}$ (above) or $TCRb^{high}CD4^{-}CD8^{+}$ (below) populations from WT or FoxN1MycTg mice. Bar graphs show mean ± SEM for a minimum n=3 mice per genotype. A two-tailed unpaired Student's t test was performed to determine significance. *p < 0.05, **p < 0.01, ***p < 0.001. The source data underlying Supplementary Figures 4c, 4d, 4e and 4f are provided as a Source Data file.

Supplementary Figure 5

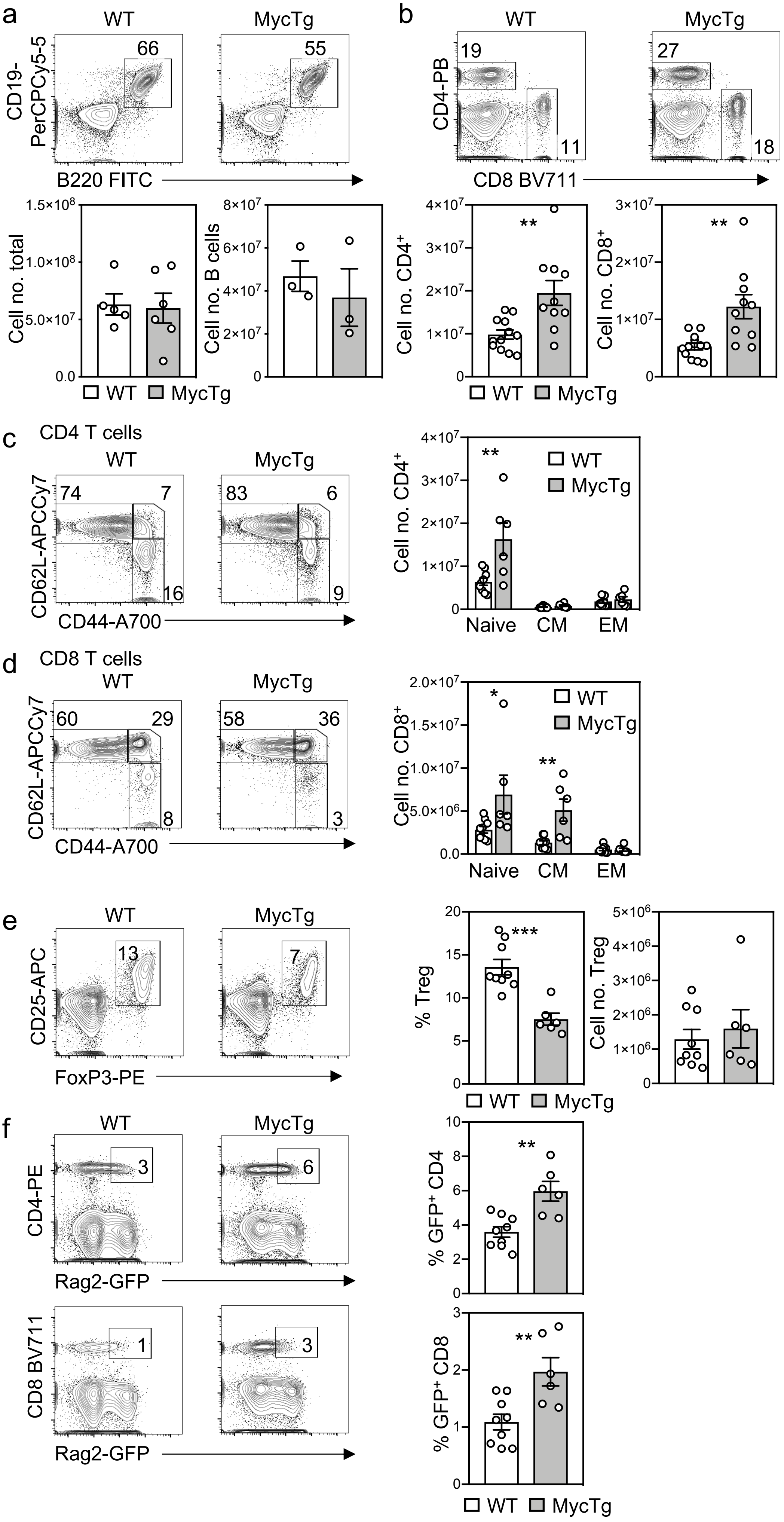

Supplementary Figure 5: Transgenic expression of Myc in TEC increases thymic function. (a) Representative flow plots of CD19 versus B220 expression on splenocytes isolated from WT or FoxN1MycTg adult mice. Bar graphs of the cell numbers of total spleen (left) or $CD19^{+}$ $B220^{+}$ B cells (right) from WT or FoxN1MycTg adult mice. (b) Representative flow plots of CD4 versus CD8 expression on splenocytes isolated from WT or FoxN1MycTg mice. Bar graphs of the cell numbers of $TCRb^{high}CD4^{+}$ $CD8^{-}$ T cells or $TCRb^{high}CD4^{-}CD8^{+}$ T cells of WT or FoxN1MycTg adult mice. (c-d) Representative flow plots of CD62L versus CD44 expression, or bar graphs of the numbers of $CD62L^{+}CD44^{-}$ naïve, $CD62L^{+}CD44^{+}$ central memory or $CD62L^{-}CD44^{+}$ effector memory populations of $TCRb^{high}CD4^{+}$ $CD8^{-}$ T cells (c) or $TCRb^{high}CD4^{-}CD8^{+}$ T cells (d) from the spleen of WT or FoxN1MycTg adult mice. (e) Representative flow plots of CD25 versus FoxP3 expression or bar graphs of the frequency and numbers of $CD25^{+}$ $FoxP3^{+}$ $TCRb^{high}CD4^{+}$ $CD8^{-}$ T Regulatory cells from the spleens of WT or FoxN1MycTg mice. (f) Representative flow plots of CD4 or CD8 versus Rag2-GFP or bar graphs of the frequency of Rag2-GFP^{+} $TCRb^{high}CD4^{+}$ $CD8^{-}$ T cells (above) or Rag2-GFP^{+} $TCRb^{high}CD4^{-}CD8^{+}$ T cells (below) in the spleen of WT Rag2-GFP or FoxN1MycTg Rag2-GFP adult mice. All bar graphs show mean ± SEM for a minimum n =3 mice per WT (white bars) or FoxN1MycTg (gray) genotype. A two-tailed unpaired Student’s t test was performed to determine significance. *$p < 0.05$, **$p < 0.01$, ***$p < 0.001$. The source data underlying Figures are provided as a Source Data file.

Supplementary Figure 6

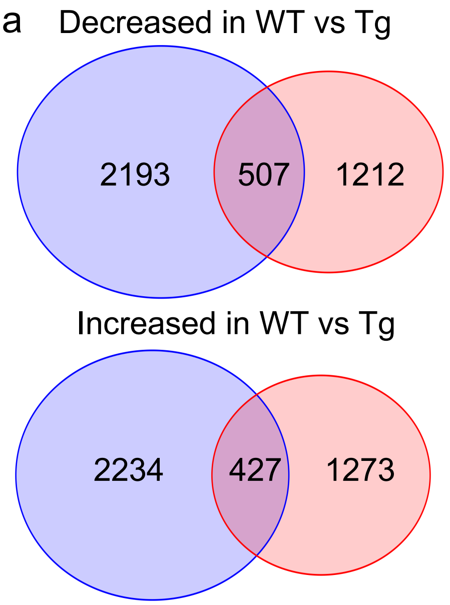

b

cTEC: Decreased in WT vs Tg

Pathway	P value
Ribosomal biogenesis	5E-20
KEGG Ribosome	5.2E-30
Translation	3E-31
RNA processing	5.4E-26

mTEC: Decreased in WT vs Tg

Pathway	P value
KEGG Ribosome	3.7E-32
Peptide chain elongation	1.3E-33
Translation	1.4E-32
rRNA processing	2.2E-20

cTEC: Increased in WT vs Tg

Response to cytokine	7.2E-6
Regulation of cell adhesion	8.2E-5
Regulation of Antigen processing and presentation	2.2E-2
Interferon gamma response	6.2E-10

mTEC: Increased in WT vs Tg

Interferon gamma response	3.4E-14
Regulation of cell-cell adhesion	1.9E-6
Response to cytokine	7.2E-6
Regulation of Antigen processing and presentation	2.3E-2

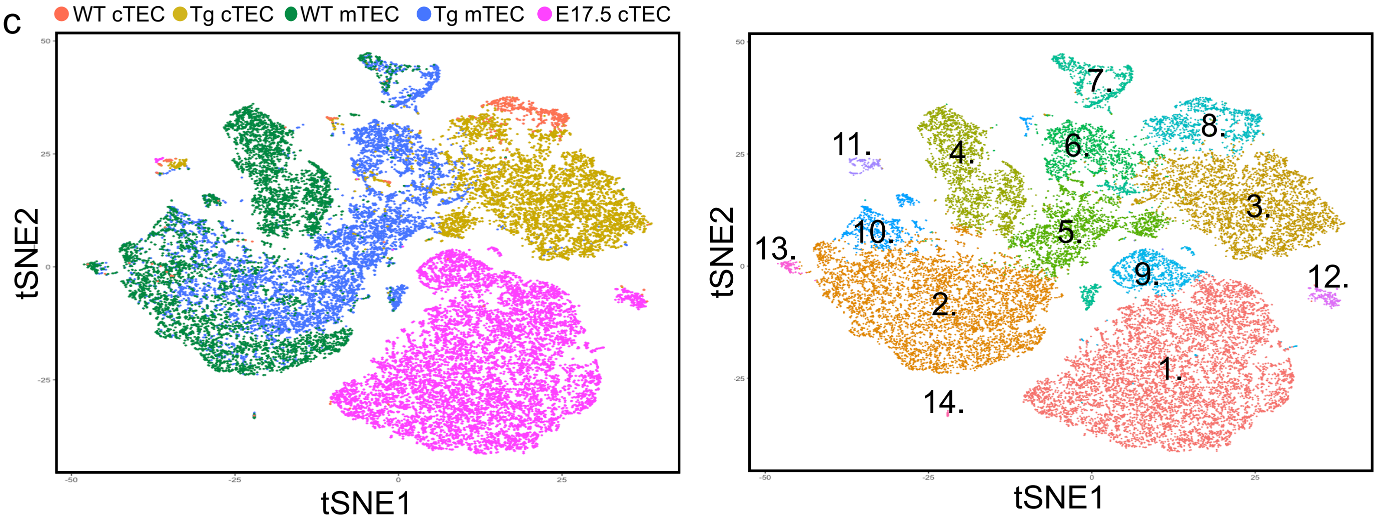

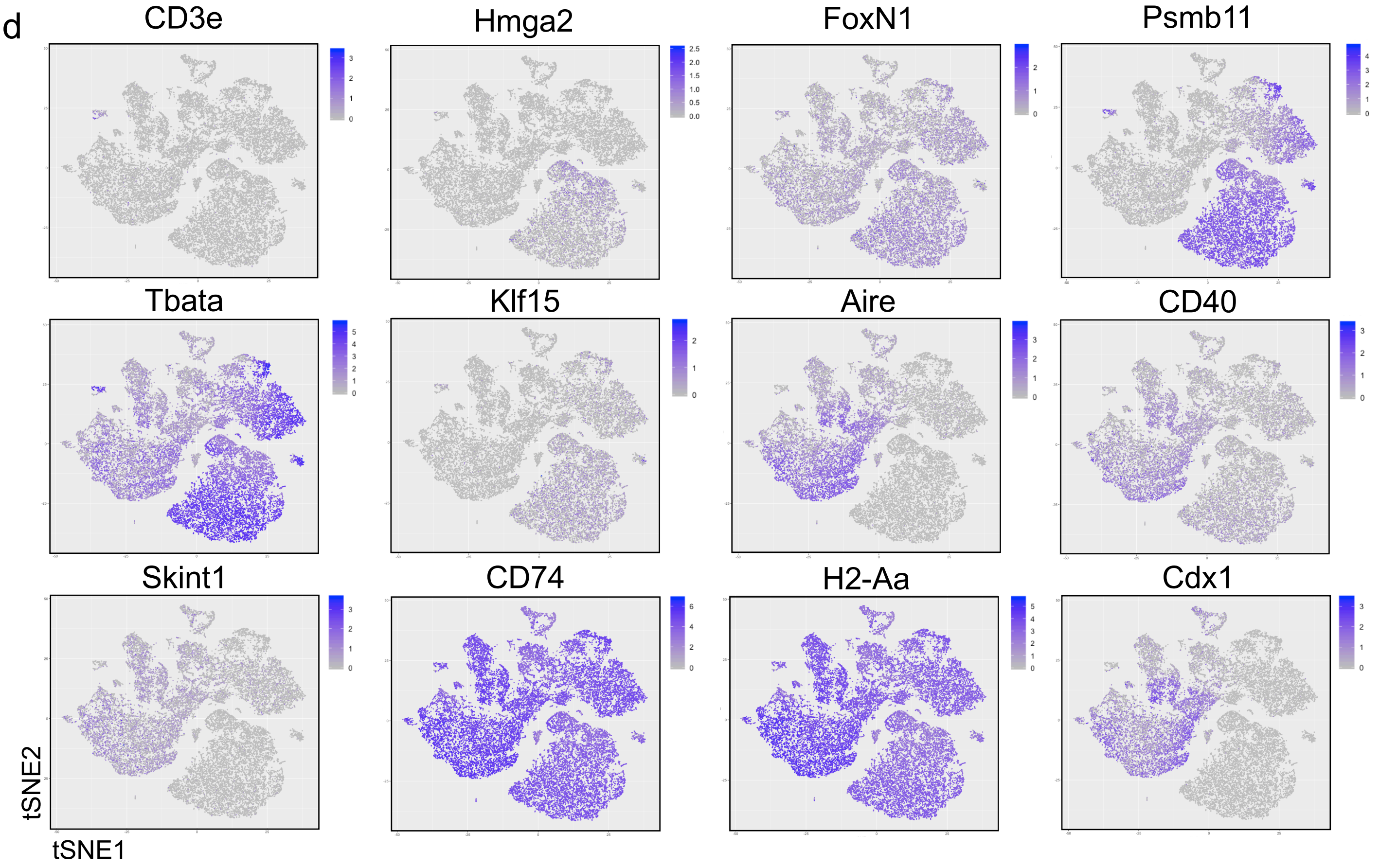

Supplementary Figure 6: Transcriptional profiling of FoxN1MycTg adult cTEC and mTEC. (a) Venn diagrams displaying the number of differentially expressed genes decreased (above) or increased (below) in adult WT cTEC (red) or mTEC (blue) compared to the equivalent FoxN1MycTg populations, derived using 2-fold change and $p<0.05$. (b) Tables of pathway enrichment analysis performed on the top 1000 genes (sorted by t-statistic) decreased (above) or increased (below) in adult WT cTEC (left) or mTEC (right) compared to FoxN1 TEC cTEC or mTEC populations, with pathways on left and p values on right. (c) A tSNE plot of scRNA-seq data performed on cTEC ($CD45^{-}EpCAM^{+}Ly51^{+}UEA^{-}$) or mTEC ($CD45^{-}EpCAM^{+}Ly51^{-}UEA^{+}$) isolated from adult WT or FoxN1MycTg mice, and a cTEC sample from embryonic day 17.5 (E17.5 cTEC), colored and clustered by identity (left) or gene expression similarity at a resolution of 0.3 (right). Each dot represents one cell. (d) tSNE plots of expression of individual genes indicated.

Supplementary Figure 7

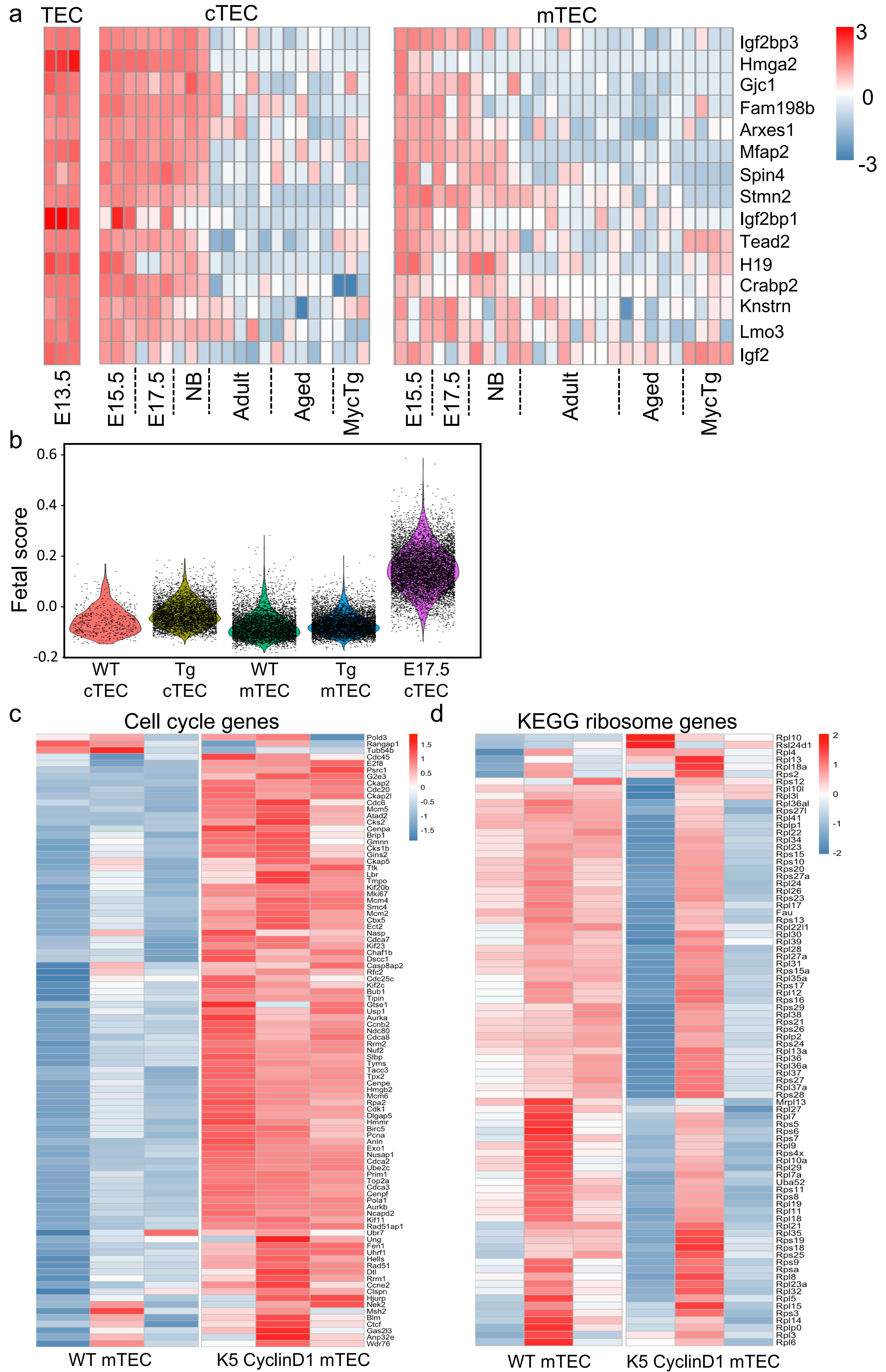

Supplementary Figure 7: Myc independent fetal signatures. (a) A heatmap of individual genes highly expressed in fetal WT TEC compared to adult WT TEC samples and not increased in expression in the adult FoxN1MycTg TEC, generated from bulk RNA-seq data, where each column displays each biological replica for its allocated time point. (b) A violin plot of a fetal score applied to the scRNA-seq data from cTEC ($CD45^{-}EpCAM^{+}Ly51^{+}UEA^{-}$) or mTEC ($CD45^{-}EpCAM^{+}Ly51^{-}UEA^{+}$) isolated from adult WT or FoxN1MycTg mice, and E17.5 cTEC; each dot represents one cell. (c-d) A heatmap on bulk RNA-seq data performed on WT and K5 Cyclin D1 adult mTEC populations for genes involved in cell cycle (c) or KEGG ribosome genes (d). Each column per genotype represents one biological replica. All heat maps in this figure are row normalized. The appearance of the middle sample in K5 Cyclin D1 transgenic mTEC for KEGG ribosome genes is an artefact of the row-normalization.

Supplementary Figure 8

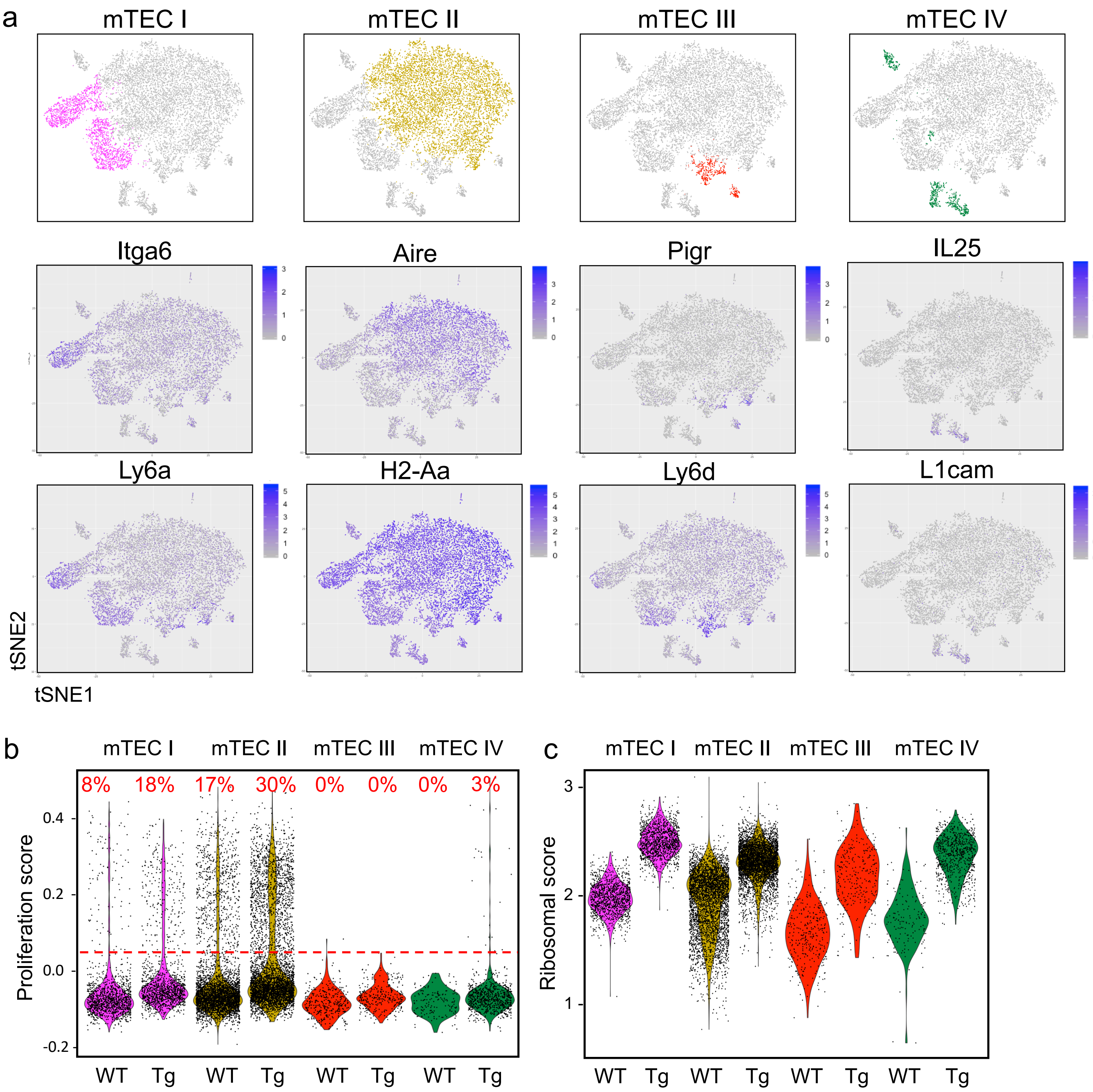

Supplementary Figure 8: The mTEC heterogeneity of FoxN1MycTg thymi. (a) tSNE plots of four individual mTEC subpopulations (above), each consisting of WT and FoxN1MycTg cells, clustered based on transcriptional similarity and labeled based on their expression of indicated signature gene expression (below). (b-c) Violin plots of the proliferation score (b) or ribosomal score (c) applied to either the WT or FoxN1MycTg cells of each of the four indicated clusters. Each dot represents one cell. The red line separates cells based on their proliferation score, high score above the line and low score below the line, percentages of cells above the line are indicated in red.
