# Supplementary material for: Myc controls a distinct transcriptional program in fetal thymic epithelial cells that determines thymus growth
Source: Nat Commun. 2019 Dec 2;10:5498. doi: 10.1038/s41467-019-13465-y (PMC6889275; doi:10.1038/s41467-019-13465-y)
Supplement: Supplementary file 3 — Reporting Summary [file 41467_2019_13465_MOESM3_ESM.pdf]

nature research

Corresponding author(s): Avinash Bhandoola

Last updated by author(s): September 24, 2019

Reporting Summary

Nature Research wishes to improve the reproducibility of the work that we publish. This form provides structure for consistency and transparency in reporting. For further information on Nature Research policies, see Authors & Referees and the Editorial Policy Checklist.

Statistics

For all statistical analyses, confirm that the following items are present in the figure legend, table legend, main text, or Methods section.

n/a	Confirmed	
☐	☒	The exact sample size (*n*) for each experimental group/condition, given as a discrete number and unit of measurement
☐	☒	A statement on whether measurements were taken from distinct samples or whether the same sample was measured repeatedly
☐	☒	The statistical test(s) used AND whether they are one- or two-sided<br>*Only common tests should be described solely by name; describe more complex techniques in the Methods section.*
☒	☐	A description of all covariates tested
☐	☒	A description of any assumptions or corrections, such as tests of normality and adjustment for multiple comparisons
☐	☒	A full description of the statistical parameters including central tendency (e.g. means) or other basic estimates (e.g. regression coefficient) AND variation (e.g. standard deviation) or associated estimates of uncertainty (e.g. confidence intervals)
☒	☐	For null hypothesis testing, the test statistic (e.g. *F*, *t*, *r*) with confidence intervals, effect sizes, degrees of freedom and *P* value noted<br>*Give P values as exact values whenever suitable.*
☒	☐	For Bayesian analysis, information on the choice of priors and Markov chain Monte Carlo settings
☐	☒	For hierarchical and complex designs, identification of the appropriate level for tests and full reporting of outcomes
☒	☐	Estimates of effect sizes (e.g. Cohen's *d*, Pearson's *r*), indicating how they were calculated

Our web collection on statistics for biologists contains articles on many of the points above.

Software and code

Policy information about availability of computer code

Data collection	Flow cytometry data was acquired on a LSRFortessa; BD using DIVA software Version 8.0.1
Data analysis	Flow cytometry analysis was done using FlowJo software (Tree Star) version 10.5.0. For Bulk RNA seq data The raw RNA-seq FASTQ reads were aligned to the mouse genome (mm10) using STAR (v. 2.5.2b) on two-pass mode with mouse Gencode (release 15) gene transfer format. For primary analysis of single cell RNA sequencing was performed with the Cellranger v2.0.1 software. Further analysis was performed using Seurat (v.2.3.4) and visualization was done using R version 3.5.3.

For manuscripts utilizing custom algorithms or software that are central to the research but not yet described in published literature, software must be made available to editors/reviewers. We strongly encourage code deposition in a community repository (e.g. GitHub). See the Nature Research guidelines for submitting code & software for further information.

Data

Policy information about availability of data

All manuscripts must include a data availability statement. This statement should provide the following information, where applicable:

- Accession codes, unique identifiers, or web links for publicly available datasets
- A list of figures that have associated raw data
- A description of any restrictions on data availability

All bulk RNA-seq data has been deposited under the GEO accession GSE129192, reviewer token: kfsroyqmjliptkb.
All scRNA-seq data has been deposited under the GEO accession GSE131339, reviewer token: klodgyichxqblqp.

Field-specific reporting

Please select the one below that is the best fit for your research. If you are not sure, read the appropriate sections before making your selection.

☒ Life sciences ☐ Behavioural & social sciences ☐ Ecological, evolutionary & environmental sciences

For a reference copy of the document with all sections, see nature.com/documents/nr-reporting-summary-flat.pdf

Life sciences study design

All studies must disclose on these points even when the disclosure is negative.

Sample size	Sample sizes were empirically determined
Data exclusions	No samples or animals were excluded from the analysis
Replication	Experiments were independently repeated to ensure the reproducibility of the findings.<br>Key findings wer validated using independent approaches to remove possible experimental bias.
Randomization	No randomization was used
Blinding	No blinding was used

Reporting for specific materials, systems and methods

We require information from authors about some types of materials, experimental systems and methods used in many studies. Here, indicate whether each material, system or method listed is relevant to your study. If you are not sure if a list item applies to your research, read the appropriate section before selecting a response.

Materials & experimental systems

n/a	Involved in the study	
☐	☒	Antibodies
☒	☐	Eukaryotic cell lines
☒	☐	Palaeontology
☐	☒	Animals and other organisms
☒	☐	Human research participants
☒	☐	Clinical data

Methods

n/a	Involved in the study	
☒	☐	ChIP-seq
☐	☒	Flow cytometry
☒	☐	MRI-based neuroimaging

Antibodies

Antibodies used	Antibodies specific for CD45.2 (104), CD45.1(A20) CD4 (GK1.5), CD8-� (53-6.72), TCR-� (H57), B220 (RA3-6B2), Aire (5H12), CD19 (1D3), Ly51 (6C3), EpCAM (G8.8), CD80 (16-10A1), MHC Class II (M5/114.15.2), CD44 (IM7), CD62L (MEL-14), CD25 (PC61.5), CD69 (H1.2F3) and Streptavidin PECy7 were from eBioscience. Biotinylated UEA-1 (B-1065) was from Vector Labs. c-Myc rabbit monoclonal antibody (cell signaling; D84C12), Goat anti Rabbit Alexa Fluor® 488 secondary (Invitrogen; A11008).
Validation	All antibodies used are commercially available, and were validated by the manufacturer. Upon receipt, antibodies were tested in the laboratory using known positive and negative controls.

Animals and other organisms

Policy information about studies involving animals; ARRIVE guidelines recommended for reporting animal research

Laboratory animals	Mice were used on a B6 background, at ages stated from embryonic mice to aged mice of 18 months+, of either sex.
Wild animals	This study does not use wild animals
Field-collected samples	This study does not use field- collected samples
Ethics oversight	The IACUC at the National Cancer Institute approved these experiments.

Note that full information on the approval of the study protocol must also be provided in the manuscript.

Flow Cytometry

Plots

Confirm that:

☒ The axis labels state the marker and fluorochrome used (e.g. CD4-FITC).

☒ The axis scales are clearly visible. Include numbers along axes only for bottom left plot of group (a 'group' is an analysis of identical markers).

☒ All plots are contour plots with outliers or pseudocolor plots.

☒ A numerical value for number of cells or percentage (with statistics) is provided.

Methodology

Sample preparation	Cells from mouse thymus or spleen were isolated by manual disruption into buffer or enzymatic digestion as noted.
Instrument	BD BioSciences LSR Fortessa, FACSAria II
Software	BD BioSciences FACSDiva for collection, Treestar Flowjo for analysis.
Cell population abundance	Thymic epithelial cells were FACS purified to above 98% purity.
Gating strategy	Cells were initially gated on size (FSC/SSC) followed by a viability dye to exclude remaining dead cells.. Plots were then gated for singlets using both SSC-H/SSC-W and FSC-H/FSC-W.

☒ Tick this box to confirm that a figure exemplifying the gating strategy is provided in the Supplementary Information.
