# Supplementary material for: Myc controls a distinct transcriptional program in fetal thymic epithelial cells that determines thymus growth
Source: Nat Commun. 2019 Dec 2;10:5498. doi: 10.1038/s41467-019-13465-y (PMC6889275; doi:10.1038/s41467-019-13465-y)
Supplement: Supplementary file 4 — Description of Additional Supplementary Files [file 41467_2019_13465_MOESM4_ESM.pdf]

Description of Additional Supplementary Files

File Name: Supplementary Data 1

Description: Lists of the top 2000 genes from the bulk RNA-seq data, expressed across all timepoints of the cTEC data set (tab one) or the mTEC data set (tab two).

File Name: Supplementary Data 2

Description: Lists of all pathways significantly altered from the GSVA analysis performed on the 2000 most differentially expressed genes across all timepoints of the cTEC data set (tab one) or the mTEC data set (tab two).

File Name: Supplementary Data 3

Description: Lists of all pathways significantly altered between WT Adult and WT Aged bulk RNA-seq data sets. Pathway enrichment analyses were performed on the top 1000 genes: increased in cTEC adult vs aged (tab one); decreased in cTEC adult vs aged (tab two); increased in mTEC adult vs aged (tab three); decreased in mTEC adult vs aged (tab four).

File Name: Supplementary Data 4

Description: Lists of all differentially expressed genes between clusters of the same developmental time point, defined in Supplementary Figure 2b. E13.5 cluster 3 vs E13.5 cluster 9 (tab one); E15.5 cluster 5 vs E15.5 cluster 2 (tab two); Newborn cluster 8 vs Newborn cluster 1 (tab 3); 4 week cluster 13 vs 4 week cluster 7 (tab four). Genes upregulated in the first cluster have a positive log fold change in column C and genes upregulated in the second cluster have a negative log fold change in column C. p-values were determined using the Wilcoxon ranked sum test and genes with a Bonferonni-adjusted p-value > 0.05 were discarded.

File Name: Supplementary Data 5

Description: Lists of all pathways significantly altered in the bulk RNA-seq data set between WT and MycTg adult TEC populations. Pathway enrichment analyses were performed on the top 1000 genes: decreased in WT cTEC vs MycTg cTEC (tab one); increased in WT cTEC vs MycTg cTEC (tab two); decreased in WT mTEC vs MycTg mTEC (tab three); increased in WT mTEC vs MycTg mTEC (tab four).

File Name: Supplementary Data 6

Description: A list of all genes differentially expressed between FoxN1MycTg cTEC populations in cluster 3 versus cluster 8, defined in Supplementary Figure 6c. Genes upregulated in cluster 3 relative to cluster 8 have a positive log fold change in column C and genes upregulated in cluster 8 relative to cluster 3 have a negative log fold change

in column C. p-values were determined using the Wilcoxon ranked sum test and genes with a Bonferonni-adjusted p-value > 0.05 were discarded.

File Name: Supplementary Data 7

Description: Lists of the most differentiated genes per mTEC cluster defined in Figure 5e: mTEC I (tab one); mTEC II (tab two); mTEC III (tab three); mTEC IV (tab four); mTEC V (tab five). Genes upregulated in the referenced mTEC subpopulation are noted by a positive average log fold change in column C and genes downregulated in the referenced mTEC subpopulation are noted by a negative average log fold change in column C. p-values were determined using the Wilcoxon ranked sum test and genes with a Bonferonni-adjusted p-value > 0.05 were discarded.

File Name: Supplementary Data 8

Description: Lists of the most differentiated genes expressed between the WT and MycTg cells that make up each of the five mTEC clusters defined in Figure 5e: mTEC I (tab one); mTEC II (tab two); mTEC III (tab three); mTEC IV (tab four); mTEC V (tab five). Genes upregulated in WT with respect to MycTg are noted by a positive log fold change in column 3, and genes downregulated in WT with respect to MycTg are noted by a positive log fold change in column 3. p-values were determined using the Wilcoxon ranked sum test and genes with a Bonferonni-adjusted p-value > 0.05 were discarded.

File Name: Supplementary Data 9

Description: A list of the 292 T cell contaminant genes that were removed from all samples prior to analysis for gene expression changes on the bulk RNA-seq data set.
